# Cellular niches for hematopoietic stem cells in bone marrow under normal and malignant conditions

**DOI:** 10.1186/s41232-023-00267-5

**Published:** 2023-02-21

**Authors:** Yoshiki Omatsu

**Affiliations:** Laboratory of Stem Cell Biology and Developmental Immunology, Graduate School of Frontier Biosciences and Graduate School of Medicine, Osaka University, 1-3 Yamada-oka, Suita, Osaka, 565-0871 Japan

**Keywords:** Hematopoietic microenvironment, Niche, Bone marrow, CAR cells

## Abstract

Throughout adult life, most lineages of blood cells, including immune cells, are generated from hematopoietic stem cells (HSCs) in the bone marrow. HSCs are thought to require special microenvironments, termed niches, for their maintenance in the bone marrow; however, the identity of the HSC cellular niche has been a subject of long-standing debate. Although diverse candidates have been proposed so far, accumulated studies demonstrate that the bone marrow-specific population of fibroblastic reticular cells with long processes, termed CXC chemokine ligand 12-abundant reticular cells (which overlap strongly with leptin receptor-expressing cells), termed CAR/LepR^+^ cells, are the pivotal cellular component of niches for HSCs and lymphoid progenitors. Sinusoidal endothelial cells (ECs) are also important for hematopoietic homeostasis and regeneration. Hematopoiesis is altered dynamically by various stimuli such as inflammation, infection, and leukemia, all of which affect cellular niches and alter their function. Therefore, it is important to consider situations in which stimuli affect HSCs, either via direct interaction or indirectly via the hematopoietic niches. In this review, the dynamics of cellular niches in the steady state and disease are described, with a focus on CAR/LepR^+^ cells and ECs.

## Background

Most types of blood cells, including immune cells, are generated from hematopoietic stem cells (HSCs) in the bone marrow. Early studies show that bone marrow-derived stromal cells support granulopoiesis and B lymphopoiesis when cultured for a long period, suggesting the existence of cellular niches for HSCs in the bone marrow [1, 2]. However, the identity of the niche has long been unclear. Bone-lining osteoblasts were the first cells proposed to be cellular niches for HSC [3, 4]. Then, diverse candidate cells for HSC niches, such as sinusoidal endothelial cells (ECs) [5], CXC chemokine ligand 12 (CXCL12)-abundant reticular (CAR) cells [6, 7], GFAP^+^ Schwann cells [8], macrophages [9, 10], Tregs [11], NG2^+^ periarteriolar cells [12, 13], and megakaryocytes [14, 15], had been reported so far. Simultaneously, the importance of these candidates had been confirmed or doubted by many studies [16–18]. For example, with several lines of negative evidence [19–21], osteoblasts have now lost their basis as HSC niches.

Accumulated series of evidence demonstrate that CAR cells, which overlap strongly with leptin receptor (LepR)-expressing cells, are the major cellular component of niches for hematopoietic stem and progenitor cells (HSPCs) [6, 7, 22–24]. Sinusoidal endothelial cells are also important for hematopoietic homeostasis and regeneration [22, 25]. High-throughput gene expression analysis at the single-cell level, which has developed rapidly in recent years, allows comprehensive analysis of hematopoietic cells and their cellular niches in the bone marrow. Furthermore, it has been clear that infection, inflammation, and leukemia lead to dynamic changes in the cellularity and properties of these cellular niches, as well as the HSPCs themselves.

### CAR/LepR^+^ cells

Stem cell factor (SCF), CXCL12, and thrombopoietin (TPO) are essential cytokines involved in maintenance of HSCs. Mice deficient in any of these genes show a marked reduction in the number of HSCs in the bone marrow [26–30]. These facts suggest that cells producing these cytokines are an important component of the HSC niche. However, of these three cytokines, TPO is the exception because it is synthesized mainly outside the bone marrow and acts as a hormone. TPO is expressed by hepatocytes, and its deletion from hepatocytes depletes HSCs from the bone marrow [31, 32].

To identify cellular niches for HSCs in the bone marrow, Sugiyama et al. generated mice in which a green fluorescent protein (GFP) reporter gene was knocked into the CXCL12 locus (CXCL12-GFP mice); they then identified a population of reticular cells with long processes that express much higher levels of CXCL12 than any other types of cells in the bone marrow and other organs. These cells were named CXCL12-abundant reticular (CAR) cells [6]. Furthermore, short-term ablation of CAR cells in vivo, using mice in which a transgene encoding the diphtheria toxin receptor (DTR) was knocked into the CXCL12 locus (CXCL12-DTR mice), led to a marked reduction of CXCL12 and SCF in the bone marrow, indicating that CAR cells are the major producer of CXCL12 and SCF in the bone marrow [7]. Subsequently, Ding et al. generated mice in which GFP was knocked into the SCF locus (SCF-GFP mice), and showed that these cells express much higher levels of SCF (overlapping strongly with CAR cells); in addition, most of these cells express much higher levels of LepR than other types of bone marrow cell [22]. Therefore, these cells will be referred to as CAR/LepR^+^ cells in the following. Recent single-cell RNA sequencing studies also revealed that CAR/LepR^+^ cells are a distinct cell population that expresses high levels of SCF and CXCL12 [33–35].

Méndez-Ferrer et al. reported Nestin-GFP positive cells as HSC niches by using Nestin-GFP transgenic mice, in which GFP is expressed under the control of the neural-specific regulatory elements of Nestin gene [36]. It is now known that Nestin-GFP^high^ cells and Nestin-GFP^low^ cells are distinct cells; Nestin-GFP^low^ cells strongly overlap with CAR/LepR^+^ cells, and Nes-GFP^high^ cells overlap with Myh11^+^ NG2^+^ cells in the periarterial area [13, 36, 37]. It is noted that adult CAR/LepR^+^ cells or Nestin-GFP^low^ cells do not express endogenous Nestin [12, 37]. In addition, Nestin-Cre or NG2-Cre label both Nestin-GFP^high^ and Nestin-GFP^low^ (or CAR/LepR^+^) cells, but NG2-CreER and Myh11-CreERT2 label peri-arterial NG2^+^ cells but not CAR/LepR^+^ cells [13, 22, 37]. Some osteoblastic lineage Cre-lines, such as Sp7-Cre, Ocn-Cre, and Dmp1-Cre, also label varying parts of CAR/LepR^+^ cells [38, 39]. Adipoq-Cre labels adipocytes and probably most CAR/LepR^+^ cells, while Adipoq-CreER labels adipocytes and a few CAR/LepR^+^ cells [40, 41].

Selective ablation of CAR/LepR^+^ cells using CXCL12-DTR mice [7] or mice in which DTR is expressed by LepR^+^ cells [37] leads to a decrease in the number of HSPCs. Similarly, when SCF is deleted from LepR^+^ cells, the number of HSPCs in the bone marrow decreases markedly [22, 42]. Interleukin-7 (IL-7) is also expressed by most CAR/LepR^+^ cells, and its deletion from CAR/LepR^+^ cells leads to a severe reduction in the numbers of pro-B and pre-B cells in the bone marrow [43]. Taken together, these results indicate that CAR/LepR^+^ cells play a critical role in maintaining not only HSCs but also B cell progenitors.

**Fig. 1:**
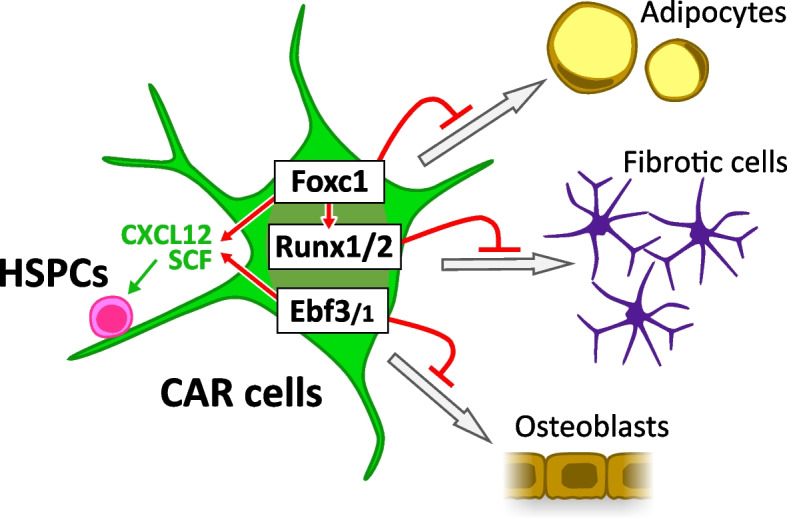
Essential transcription factors in CAR/LepR^+^ cells

Foxc1 was first identified as an essential transcription factor that is preferentially expressed by CAR cells in the bone marrow. When Foxc1 is conditionally deleted from CAR/LepR^+^ cells, the number of HSPCs is markedly reduced, accompanied by a marked increase in the number of adipocytes in the bone marrow [23]. These results indicate that Foxc1 is essential for the maintenance of niches for HSPCs and for inhibiting differentiation of CAR cells into adipocytes (Fig. 1). Another transcription factor, Ebf3, is also expressed preferentially by CAR cells. Ebf1 is expressed by B cell precursors and is critical for B cell development; however, it is expressed preferentially by CAR cells among all bone marrow non-hematopoietic cells. When both Ebf3 and Ebf1 are deleted from CAR/LepR^+^ cells, the number of HSPCs in the bone marrow is markedly reduced, accompanied by a marked increase of trabecular bones [24]. These results indicate that Ebf1/3 play an essential role in the maintenance of HSPC niches and the bone marrow cavity by inhibiting the differentiation of CAR cells into osteoblasts. Transcription factor Snai2 is also expressed at high levels by CAR/LepR^+^ cells, and Snai2 null mice (129Sv/C57BL/6 F1 background) or conditional knockout mice (Mx1-Cre; Snai2-flox) show slightly decreased HSC numbers in the bone marrow. It is argued that increased expression of osteopontin (Spp1) may be responsible for the phenotype, but precise verification using CAR/LepR^+^ cell-specific knockout mice would be needed to confirm this [44]. A more recent study showed that transcription factors Runx1 and Runx2 are expressed at high levels by CAR cells and that the bone marrow is markedly fibrotic and hematopoiesis is severely impaired in Runx1 and Runx2 conditional knockout mice. These results indicate that CAR cells require Runx1 or Runx2 to prevent their fibrotic conversion and to maintain HSCs and hematopoiesis in adults [45]. Elucidation of the interactions and functional links among these transcription factors will be the subject of future studies.

### Sinusoidal endothelial cells

Bone marrow venous endothelial cells, termed sinusoidal endothelial cells, are thought to be a component of the HSPC niche because they promote the maintenance of HSPCs in culture [25, 46]. Bone marrow endothelial cells express low levels of SCF and CXCL12, which are required for the maintenance and regeneration of HSPCs [7, 33–35]. Conditional deletion of either of these factors from endothelial cells decreases the number of HSPCs in the bone marrow [22]. However, it remains unclear whether endothelial cells synthesize these factors entirely in the bone marrow, or whether endothelial cells outside the bone marrow contribute to their production. In the fetal stage when HSCs proliferate in the liver, SCF derived from hepatic stellate cells is important, but EC-derived SCF also shows some contribution [47]. The sinusoidal endothelium might be essential for hematopoietic homeostasis due to its function as a regulated gate for differentiated blood cells, rather than as a source of hematopoietic cytokines. Furthermore, sinusoidal endothelial cells appear to play an important role in the regeneration of hematopoiesis after bone marrow destruction [25, 48].

Bone marrow endothelial cells are divided into subpopulations based on differential expression of PECAM-1 (CD31) and endomucin (Emcn). Type-H (CD31^hi^ Emcn^hi^) endothelial cells are tightly coupled to osteogenesis and localize in the metaphysis and endosteum of postnatal long bone [49, 50]. Notch signaling and integrin-β1 expression by endothelial cells are required for the specificity and function of Type-H subpopulations associated with osteoprogenitors [50, 51]. However, the molecular basis of Type-L (CD31^lo^ Emcn^lo^) endothelial cells, which comprise the majority of the bone marrow space during hematopoiesis, is largely unknown.

### The bone marrow microenvironment at single-cell resolution

In recent years, a series of reports have undertaken comprehensive transcriptome analysis of non-hematopoietic cells in the bone marrow at the single-cell level. These studies confirmed that CAR/LepR^+^ cells are the major producers of hematopoietic cytokines, suggesting a minimal contribution by other non-hematopoietic cells such as endothelial cells, osteoblasts, and nerve cells [33–35]. It is also suggested that the CAR/LepR^+^ cell cluster, which shows uniform expression of key niche factor genes such as *Cxcl12*, *Kitl*, *Foxc1*, and *Ebf3*, may be divided into several subclusters based on the expression of cell differentiation marker genes. Although these subclusters are likely to reflect their cellular origin or some bias in differentiation potential, their boundaries are ambiguous. Their specific functions and potentials should be fully validated by subcluster-specific depletion and/or tracing. For example, CAR/LepR^+^ subpopulations that produce high levels of osteolectin localize near the endosteum and periarterial areas, and they are suggested to be particularly supportive of B-cell development [52]. Further studies are needed to identify differences in cell lineages, as well as the specific functions of these subpopulations of CAR/LepR^+^ cells.

Furthermore, these comprehensive analyses revealed dynamic changes in the proportions of cell clusters, as well as gene expression, in bone marrow non-hematopoietic cell populations during stresses such as 5-FU administration [33] and leukemia [34]. In the future, these analyses at single-cell resolution will provide new findings that go beyond confirming the results of conventional methods. However, it should be noted that single-cell analysis may not include all cells due to technical limitations. For example, macrophage populations in the bone marrow are difficult to isolate without enzymatic treatment, meaning that macrophage-derived cell fragments and mRNA can contaminate other cell populations [53].

### The niche changes in response to inflammation, infection, and leukemia

**Fig. 2:**
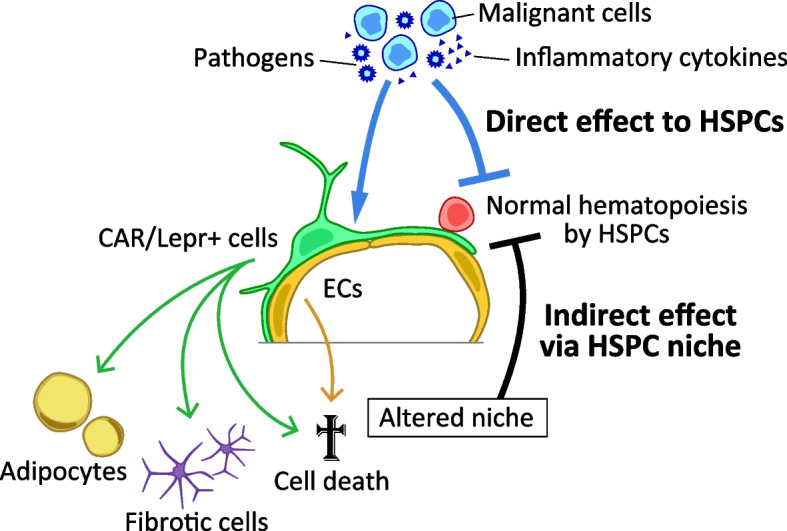
Two pathways that impair normal hematopoiesis upon inflammation, infection, or leukemia

HSPCs can sense and respond to pathogen-derived molecules and inflammatory cytokines directly [54, 55]. On the other hand, the bone marrow microenvironment also alters its niche function by sensing stimuli such as pathogens, inflammatory cytokines, and leukemic cells [16, 56, 57]. Therefore, it is necessary to consider cases in which those stimuli affect HSPCs directly, and when they affect HSPCs indirectly via the hematopoietic niches (Fig. 2).

Endothelial cells express several pattern recognition receptors (PRRs) such as toll-like receptors (TLRs), which sense pathogens, and activate HSPCs to proliferate and accelerate myelopoiesis (emergency hematopoiesis). For example, expression of TLR4 and MyD88 by endothelial cells is required for neutrophil recruitment in response to lipopolysaccharides (LPS) derived from *Escherichia coli*, and subsequent secretion of granulocyte colony-stimulating factor (G-CSF) initiates emergency hematopoiesis [58].

Mesenchymal cell populations, including CAR/LepR^+^ cells, also recognize surrounding inflammatory signals that occur locally or systemically during infection and inflammation. Activation of various PRRs leads to the production of additional factors and alters the expression of cytokines and chemokines in cellular niches. Studies using models of acute viral infection suggest that mesenchymal cells are stimulated by interferon-γ to secrete interleukin-6 (IL-6), leading to the expansion of myeloid progenitors and mature myeloid cells [59]. Similarly, in chronic repetitive LPS-induced inflammation, CAR cells are the main producers of IL-6, which is important for sustained myeloid production [60]. Furthermore, upregulation of G-CSF during infection is thought to promote myeloid hematopoiesis and suppress CXCL12 production by CAR cells, resulting in the mobilization of HSPCs to the circulation [61]. In addition, CAR/LepR^+^ cells increase the number of circulating monocytes by upregulating the expression of the migratory chemokine CCL2, which is important for efficient clearance of *Listeria monocytogenes* infection [62].

Radiation and chemotherapies, such as 5-fluorouracil (5-FU), cause myeloablation, an increase in adipocytes, and damage to the vascular network [33, 41, 48]. The mechanisms underlying endothelial cell regeneration after injury are at least partially related to VEGFR2 signaling [48]. Furthermore, the transplantation of granulocytes into irradiated mice enhances endothelial cell regeneration via TNF-α signaling [63]. These studies on dynamics, as well as studies that identify essential signals for endothelial cells and CAR/LepR^+^ cells during the regeneration process, will have important implications for clinical applications.

Many studies report changes in the bone marrow environment during leukemia. It has become clear that leukemic cells can alter the hematopoietic niche in a variety of ways and disrupt homeostasis of hematopoietic cells. Studies in a mouse model of myeloproliferative neoplasms such as chronic myeloid leukemia (CML) revealed that malignant cells transform osteoblast lineage cells (maybe including CAR/LepR^+^ cells) into inflammatory myelofibrotic cells, supporting malignant cells at the expense of normal hematopoiesis [64]. In addition, it has been reported that a major cause of hematopoietic defect is IL-6 produced by CML cells [65], which impacts both HSPC and their cellular niches. Indeed, HSPC niche function is impaired including decreased expression of CXCL12 in CAR cells in the CML mice [66, 67]. These results were confirmed in recently identified human CAR cells from CML patients [68]. Furthermore, thymidine kinase inhibitor (TKI)-resistant leukemic stem cells (LSCs) in CML are maintained in the bone marrow in a CXCL12-dependent manner, indicating that loss of CXCL12 inhibits maintenance of LSC quiescence and makes them sensitive to TKIs [67]. Primary myelofibrosis (PMF) is associated with bone marrow fibrosis; mouse models show that transforming growth factor-beta (TGF-β) and platelet-derived growth factors (PDGFs) are involved in fibrosis and that the source of myofibroblasts is CAR/LepR^+^ cells [69]. It has also been shown that altered cellular niches produce alarmins such as S100A8/A9 that accelerate fibrosis in PMF model mice [70]. Interestingly, in myelodysplastic syndrome (MDS) model mice, the involvement of extracellular vesicles containing microRNAs has been suggested as leukemic cell-derived factors that suppress the niche function for normal hematopoiesis [71].

Thus, the bone marrow hematopoietic environment is thought to be altered differently by inflammation, infection, or leukemia. These changes lead to differences in hematopoietic activity and/or resistance to therapy. In the steady state, CAR/LepR^+^ cells act as central cellular niches, but this is not always the case in disease. Certain malignant cells that actively impair hematopoiesis may emerge during disease states. To understand precisely the heterogeneous hematopoietic environment in disease conditions, comprehensive single-cell level analysis can be a powerful tool [33, 34, 70]. These analyses have been expected to reveal the key cellular components controlling each disease as therapeutic targets. Further studies will be needed to reveal the actual effector cells involved and the essential molecular mechanisms underlying each phenomenon.

## Conclusions

There is accumulating evidence for a hematopoietic microenvironment in the bone marrow. In particular, comprehensive analysis at the single-cell level has confirmed that CAR/LepR^+^ cells play a pivotal role in the HSPC niche as they produce the greatest amounts of cytokines that are essential for hematopoiesis.

Inflammation, infection, and leukemia have a significant effect on CAR cells and endothelial cells, as well as HSPCs, leading to altered functions. Depending on the situation, the actual effector cells and the molecular mechanisms might be diverse, and many are not fully understood. In addition, we do not really know how long changes in the niche persist, or how the cellular niches and normal hematopoietic cells regenerate. Because various hematopoietic and non-hematopoietic cells can be effectors and/or mediators, the dynamics are quite complex. Thus, a comprehensive view of all cells in the bone marrow is needed. It is important to identify key cell types and key signaling pathways that function during changes in the bone marrow because they may be clinical targets for controlling hematopoietic homeostasis.

## Data Availability

Not applicable.

## Abbreviations

HSC: Hematopoietic stem cell
CAR: CXCL12-abundant reticular
EC: Endothelial cell
HSPC: Hematopoietic stem/progenitor cell
GFAP: Glial fibrillary acidic protein
SCF: Stem cell factor
TPO: Thrombopoietin
GFP: Green fluorescent protein
DTR: Diphtheria toxin receptor
PRR: Pattern recognition receptor
LCMV: Lymphocytic choriomeningitis virus
TGF-β: Transforming growth factor-beta
PDGF: Platelet-derived growth factor
CML: Chronic myeloid leukemia
TKI: Thymidine kinase inhibitor
LSC: Leukemic stem cell
PMF: Primary myelofibrosis
